# A novel heterozygous *COL4A4* missense mutation in a Chinese family with focal segmental glomerulosclerosis

**DOI:** 10.1111/jcmm.12924

**Published:** 2016-07-29

**Authors:** Yuan Wu, Pengzhi Hu, Hongbo Xu, Jinzhong Yuan, Lamei Yuan, Wei Xiong, Xiong Deng, Hao Deng

**Affiliations:** ^1^ Center for Experimental MedicineThe Third Xiangya HospitalCentral South UniversityChangshaChina; ^2^ Department of NeurologyThe Third Xiangya HospitalCentral South UniversityChangshaChina; ^3^ Department of Clinical LaboratoryThe Third Xiangya HospitalCentral South UniversityChangshaChina; ^4^ Department of RadiologyThe Third Xiangya HospitalCentral South UniversityChangshaChina; ^5^ Department of NephrologyThe Third Xiangya HospitalCentral South UniversityChangshaChina; ^6^ Cancer Research InstituteXiangya School of MedicineCentral South UniversityChangshaChina

**Keywords:** focal segmental glomerulosclerosis, the *COL4A4* gene, collagen IV nephropathies, exome sequencing, mutation

## Abstract

Focal segmental glomerulosclerosis (FSGS) is the most common glomerular histological lesion associated with high‐grade proteinuria and end‐stage renal disease. Histologically, FSGS is characterized by focal segmental sclerosis with foot process effacement. The aim of this study was to identify the disease‐causing mutation in a four‐generation Chinese family with FSGS. A novel missense mutation, c.1856G>A (p.Gly619Asp), in the collagen type IV alpha‐4 gene (*COL4A4*) was identified in six patients and it co‐segregated with the disease in this family. The variant is predicted to be disease‐causing and results in collagen IV abnormalities. Our finding broadens mutation spectrum of the *COL4A4* gene and extends the phenotypic spectrum of collagen IV nephropathies. Our study suggests that exome sequencing is a cost‐effective and efficient approach for identification of disease‐causing mutations in phenotypically complex or equivocal disorders. Timely screening for *COL4A3/COL4A4* mutations in patients with familial FSGS may help both accurately diagnose and treat these patients.

## Introduction

Focal segmental glomerulosclerosis (FSGS) is a glomerular histological lesion associated with proteinuria and end‐stage renal disease (ESRD), and the incidence is about 2.3/100,000 in general population from the United States 1, 2. FSGS is a morphological/histological pattern of injury rather than a specific glomerular disease, and it is characterized by focal [less than 50% of glomeruli was affected on light microscopy (LM)] and segmental [less than 50% of glomerular tuft was affected] glomerular sclerosis, and foot process effacement 3, 4. Genetic or non‐genetic factors may lead to FSGS 5. More than 20 mutated podocyte genes, including the transient receptor potential canonical channels type 6 gene (*TRPC6*), the alpha‐actinin‐4 gene (*ACTN4*), the CD2‐associated protein gene (*CD2AP*), the inverted formin 2 gene (*INF2*), the nephrin gene (*NPHS1*), the NPHS2 podocin gene (*NPHS2*), the Wilms tumour 1 gene (*WT1*) and the Rho GTPase‐activating protein 24 gene (*ARHGAP24*), have been identified to cause familial FSGS 6. In 2014, Malone *et al*. first identified the collagen type IV alpha‐3 gene (*COL4A3*, MIM 120070) and collagen type IV alpha‐4 gene (*COL4A4*, MIM 120131) variants in a cohort of patients with a primary diagnosis of familial FSGS, suggesting that the *COL4A3*/*COL4A4*‐caused phenotypes in mature glomerular basement membrane (GBM) may cause primary FSGS 2, 6.

In this study, we performed exome sequencing to identify the genetic cause of autosomal‐dominant FSGS in a four‐generation Chinese Han pedigree. A novel missense mutation, c.1856G>A (p.Gly619Asp), in the *COL4A4* gene was found to co‐segregate with FSGS in this family. The missense mutation may be the genetic cause of FSGS.

## Materials and methods

### Participators and clinical evaluation

**Figure 1:**
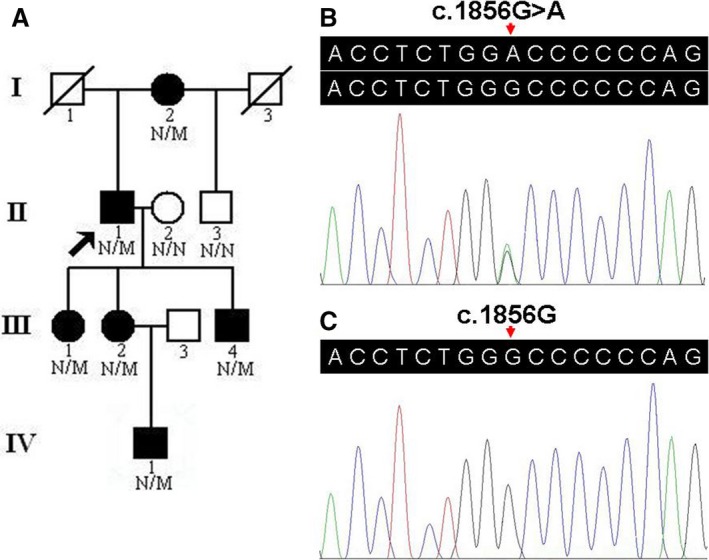
Pedigree and sequence analysis of an FSGS family. (**A**) Pedigree of the family with FSGS. N: normal; M: the *COL4A4* c.1856G>A (p.Gly619Asp) variant. Arrow indicates the proband. (**B**) Sequence of heterozygous c.1856G>A (p.Gly619Asp) variant. (**C**) Sequence of a normal control. FSGS: Focal segmental glomerulosclerosis.

A four‐generation Chinese Han pedigree consisting of 11 individuals was recruited from the Third Xiangya Hospital, Central South University, China (Fig. 1A). Clinical data and peripheral blood samples were obtained from eight members of the pedigree, including six affected individuals (I:2, II:1, III:1, III:2, III:4 and IV:1) and two unaffected members (II:2 and II:3). Blood samples were also collected from 100 unrelated ethnically matched normal controls (male/female: 50/50, age 38.5 ± 5.6 years). Written informed consent was obtained from the participating individuals or their guardians, and this study received approval from the Ethics Committee of the Third Xiangya Hospital, Central South University, China.

The diagnosis of FSGS was confirmed in the family member (III:2) based on pathological findings in biopsied kidney tissues, including the presence of focal, segmental areas of glomerular sclerosis with associated hyalinosis and adhesions of sclerotic tufts to Bowman's capsule on LM and the presence of some degree of podocyte foot process effacement as assessed by electron microscopy (EM) 2. In particular, no mesangial staining was observed with anti‐IgA antibodies. The diagnosis of familial FSGS was ascertained after two additional family members were found to have proteinuria (defined as proteinuria equal or higher than ++ or 150 mg/day) and progressive renal failure, or biopsy‐proven kidney disease. We excluded all cases of secondary FSGS caused by systemic diseases, such as obesity, hypertension and HIV infection 7.

### Exome capture

Genomic DNA (gDNA) was extracted from peripheral blood using phenol–chloroform extraction method 8. Exome sequencing of gDNA from one patient (II:1) was performed by Novogene Bioinformatics Institute, Beijing, China. Paired‐end DNA library was prepared and whole exome capture was carried out using Agilent's SureSelect Human All Exon V5 Kit (Agilent Technologies Inc., Santa Clara, CA, USA). After DNA quality assessment, captured DNA library was sequenced on a HiSeq 2000 platform to generate 100‐bp paired‐end reads according to the manufacturer's protocols (Illumina Inc., San Diego, CA, USA) 9, 10.

### Variant analysis and direct Sanger sequencing

After duplicate removal, local alignment, and base quality recalibration by Picard (http://sourceforge.net/projects/picard/), Genome Analysis Toolkit and SAMtools, the analysis‐ready BAM alignment results were obtained. The thresholds for calling single nucleotide polymorphisms (SNPs) included alignment rate of sequencing reads ≥95% and the coverage of sequence depth ≥10× 11, 12. CNVnator was utilized to do CNV detection 13. ANNOVAR (Annotate Variation) was used to annotate SNPs and insertions/deletions (indels) 14. Filtrations of all identified variations were performed with data from public databases including the single nucleotide polymorphism database (dbSNP build 137, http://www.ncbi.nlm.nih.gov/projects/SNP/snp_summary.cgi), 1000 genomes project (2012 April release, http://www.1000genomes.org/), and NHLBI exome sequencing project (ESP) 6500 15. After excluding common variants, retained variants were considered to be ‘novel’. Only SNPs occurring in exons or in canonical splicing sites were further analysed, and non‐synonymous SNPs were submitted to Sorting Intolerant from Tolerant (SIFT) and Polymorphism Phenotyping version 2 (PolyPhen‐2) for functional prediction 16, 17. The retained gene variant was prioritized for validation if the gene was related to glomerular disorders, such as primary IgA nephropathy, Alport syndrome (AS), thin basement membrane nephropathy (TBMN) and primary FSGS 18. The filtering prioritization process was conducted to identify the pathogenic variant in the proband and family, similar to those performed in recent studies 19. Direct Sanger sequencing was applied to confirm the potential pathogenic variant with ABI3500 sequencer (Applied Biosystems, Foster City, CA, USA) 19.The primer sequences used for PCR amplification and Sanger sequencing were shown as follows: 5′‐CATGGACATTCAGTGGTTGG‐3′ and 5′‐TTCTGACCCTTCAAGCCATC‐3′.

### Bioinformatics analysis of the mutation

Multiple sequence alignments among various species were conducted using the Basic Local Alignment Search Tool (http://blast.st-va.ncbi.nlm.nih.gov/Blast.cgi). MutationTaster (http://www.mutationtaster.org/) was further employed to evaluate the possible pathogenicity of amino acid substitution 20.

## Results

### Clinical characteristics of the pedigree

Six members of this family, including three males and three females, had symptomatic glomerulopathy diagnosed by two independent nephrologists. Consanguinity was denied by the family members. The main clinical manifestation was microscopic haematuria, which was present in all patients. Proteinuria was present in three of the six patients and chronic kidney disease (CKD) occurred in two of the six patients. Occurrence of the disease in all four generations and the male‐to‐male transmission pattern indicated an autosomal‐dominant inheritance mode of disease. The clinical characteristics of the pedigree are summarized in Table 1.

**Table 1:** Clinical and genetic data of six patients with *COL4A4* c.1856G>A (p.Gly619Asp) variation

Subject	I:2	II:1	III:1	III:2	III:4	IV:1
Sex	F	M	F	F	M	M
Age (years)	85	60	36	34	31	10
Genotype	Heterozygote	Heterozygote	Heterozygote	Heterozygote	Heterozygote	Heterozygote
Onset age (years)	50	50	35	33	30	10
Renal function	CKD	CKD	Normal	Normal	Normal	Normal
Microscopic haematuria	Yes	Yes	Yes	Yes	Yes	Yes
Proteinuria	Yes	Yes	No	Yes	No	No
Uraemia	No	No	No	No	No	No
Audiological examination	Normal	Normal	Normal	Normal	Normal	Normal
Ophthalmic examination	Normal	Normal	Normal	Normal	Normal	Normal

*COL4A4*: the collagen type IV alpha‐4 gene; CKD: chronic kidney disease.

### Exome sequencing

We performed exome sequencing of the proband (II:1, Fig. 1A) in the Chinese family with FSGS. About 40.47 million reads (99.92%) were mapped to the human reference genome. The average sequencing depth on target region was 73.24. Of the region, 98.80% was covered by the target sequence at 10× or greater. A total of 36,824 SNPs, including 17,749 in the exon regions and 1624 in the splicing sites, were identified. A total of 2472 indels, including 417 in the exon regions and 177 in the splicing sites, were detected.

### *COL4A4* mutation screening

A prioritization scheme similar to that described in recent studies was applied to identify the pathogenic variant in the proband. We excluded common known variants identified in public databases, including dbSNP137 (MAF > 1%), 1000 genomes project and NHLBI ESP6500. SIFT and PolyPhen‐2 analyses were used to predict the functional effects of non‐synonymous variants. Using the above filtering criteria, only 177 novel variants were identified to be possible disease‐causing variants and were prioritized for further analysis. In the proband, only a novel heterozygous variant, c.1856G>A (p.Gly619Asp) in the exon 25 of the *COL4A4* gene, was suspected to be the pathogenic variant, and no other variants in the known disease‐causing genes for glomerular disorders were identified. The variant was subsequently confirmed by Sanger sequencing and the same heterozygous variant was identified in all other affected family members (I:2, III:1, III:2, III:4 and IV:1) (Fig. 1B). The variant co‐segregated with disease in the family. The variant was absent in 100 unrelated Chinese healthy controls and public databases (Fig. 1C).

### Bioinformatics analysis of the mutation

**Figure 2:**
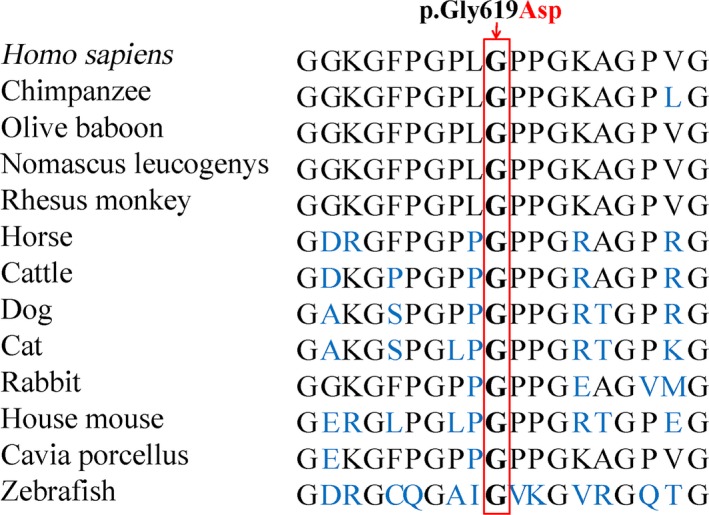
Conservation analysis of the collagen type IV α4 chain p.Gly619 amino acid residue.

The glycine at position 619 (p.Gly619) is conserved across vertebrates from human to zebrafish (Fig. 2). The SIFT prediction gained a score of 0.00, indicating that the mutation is predicted to be damaging. PolyPhen‐2 analysis of the missense mutation produced a score of 1.00 on the HumVar database (sensitivity, 0.00; specificity, 1.00), predicting it to be probably damaging. MutationTaster predicted that the substitution was disease‐causing with a probability value close to 1, which indicates a high security of prediction 21.

## Discussion

FSGS was first reported in the 1970s and it was considered as a lesion rather than a disease. FSGS accounts for approximately 20% of patients with nephrotic syndrome in children and 40% of such cases in adults 22. It can be classified as primary and secondary FSGS 1. Primary FSGS is caused by structure or function defects inherent in the podocyte 6, 23; up to 18% of primary FSGS cases is attributed to genetic mutations. Secondary FSGS is associated with a variety of causes, including viral infections, obesity, chronic hypertension, immunological processes (e.g. IgA nephropathy and immune complex nephritis) and drug abuse 24.

The *COL4A4* gene is a large gene with 48 exons and is located at chromosome 2q35‐q37. The gene encodes the α4 chain (1690 amino acid residues) of type IV collagen, a major constituent of basement membranes, which is composed of six genetically distinct α chains: two major chains α1(IV) and α2(IV), and four minor chains α3(IV), α4(IV), α5(IV) and α6(IV) 25. *COL4A3* and *COL4A4* are two genes located head to head, coding for the α3 and α4 chains of type IV collagen, respectively. The α3 and α4 chains of type IV collagen are specifically expressed in the GBM, inner ear and eye 26, 27. Mutations in the *COL4A3*/*COL4A4* gene produce abnormal α3/α4(IV) chain, which fails to incorporate properly into the triple helix of type IV collagen, and leads to destabilization of the molecular superstructure. More than 330 different mutations for the *COL4A3* gene and the *COL4A4* gene, including point mutations and complex rearrangements, have been reported both in autosomal‐recessive and autosomal‐dominant AS, as well as in TBMN 27, 28. Individuals carrying a heterozygous *COL4A3* or *COL4A4* mutation were reported to have a very different clinical outcome, ranging from being healthy to microscopic haematuria and to progressive renal failure 29.

The pathomechanism of the mutations in mature GBM collagen (IV) causing FSGS is not fully understood. Various rat models and patients of FSGS indicated injury to podocytes, and adherences of the parietal epithelial cells (PECs) to the naked GBM are the critical event in the formation of FSGS lesions 24, 30, 31. Because collagen IV α3/α4/α5 chains originate solely from podocytes, the abnormal GBM also can convey inappropriate signals to the adherent endothelial cells and podocytes, which, in turn, causes progressive disease 32. PECs proliferation was observed in crescentic glomeruli of *Col4a3*‐deficient mice 33, and the mutation in collagen IV gene may lead to activation and proliferation of PECs in FSGS. Similar to the laminin β2 gene (*LAMB2*), a FSGS gene encoding a part of one major GBM protein laminin‐521, the mutation in the *COL4A4* gene may play a direct role in the pathogenesis of FSGS 2.

Glycine substitution mutations were described in the collagen IV gene which account for 25–50% of published mutations in X‐linked and autosomal‐recessive AS, and TBMN 34. Glycine is the smallest amino acid that fits precisely into the middle of the collagen heterotrimer and the substitution may disrupt triple helix structure of the collagen network, which probably results in disease 34. In our study, we found a glycine substitution p.Gly619Asp in the *COL4A4* gene in a Chinese Han family with FSGS, and none of the patients in our family present ESRD, consistent with similar glycine substitution mutations that have been reported in patients with mild phenotypes and no renal failure 35.

Given that heterozygous *COL4A3/COL4A4* mutations were observed in patients with familial FSGS 2, 4, and FSGS can be secondary to TBMN and AS 36, 37, 38, FSGS may be only a pathological lesion process in the related kidney diseases or secondary to the GBM pathology. Therefore, *COL4A3/COL4A4*‐associated TBMN, FSGS and AS may be better classified as subtypes of collagen IV nephropathies, caused by collagen IV abnormalities, because of the clinical overlap and multiple mutations of the same gene in this group of disorders.

In our study, diagnostic biopsy in our family member (III:2) showed the typical findings of focal segmental sclerosis on LM and foot process effacement on EM, normal thickness on GBM, and absence of typical GBM morphology for TBMN or AS. However, we are unable to rule out TBMN in the early age for the 33‐year female patient, because TBMN, AS and FSGS can be caused by mutations of the collagen IV gene and all of them can result in a slow but progressive functional impairment of the podocyte and GBM, which subsequently disturbs the integrity of the whole glomerular filtration barrier and finally develops severe kidney damage 23. Patients in our family showed a wide spectrum of phenotypes, which is consistent with those of cases with the *COL4A3/COL4A4* heterozygous mutations by Heidet *et al*. 39, 40.

In conclusion, a novel missense mutation, c.1856G>A (p.Gly619Asp), in the *COL4A4* gene was identified in a Chinese Han family with FSGS. To our knowledge, this is the first report of c.1856G>A (p.Gly619Asp) in the *COL4A4* gene. Our finding as well as other investigators’ data extend the phenotypic spectrum of collagen IV nephropathies and indicate the need for new classification of glomerular disorders. *COL4A3/COL4A4*‐associated TBMN, FSGS and AS may be more appropriate to be classified as collagen IV nephropathies. Additionally, screening mutations in familial FSGS patients may play a role in accurately diagnosing and treating these patients. Further functional studies of the *COL4A3/COL4A4* mutations and application of *in vitro* and/or *in vivo* models with genetic deficiency are warranted to facilitate a better understanding of the pathogenesis and development of effective treatments for collagen IV nephropathies.

## Conflicts of interest

The authors confirm that there are no conflicts of interest.
